# Visual Robot Relocalization Based on Multi-Task CNN and Image-Similarity Strategy

**DOI:** 10.3390/s20236943

**Published:** 2020-12-04

**Authors:** Tao Xie, Ke Wang, Ruifeng Li, Xinyue Tang

**Affiliations:** 1 State Key Laboratory of Robotics and System, Harbin Institute of Technology, 92 Xidazhi Street, Harbin 150006, China; xietao@hit.edu.cn (X.T.); wangke@hit.edu.cn (K.W.); 2 MFIN, Faculty of Business and Economics, The University of Hong Kong, Pokfulam Road, Hong Kong 999077, China; txinyue@connect.hku.hk

**Keywords:** multi-task CNN, 6D relocalization, scene recognition, dual-level image-similarity strategy

## Abstract

The traditional CNN for 6D robot relocalization which outputs pose estimations does not interpret whether the model is making sensible predictions or just guessing at random. We found that convnet representations trained on classification problems generalize well to other tasks. Thus, we propose a multi-task CNN for robot relocalization, which can simultaneously perform pose regression and scene recognition. Scene recognition determines whether the input image belongs to the current scene in which the robot is located, not only reducing the error of relocalization but also making us understand with what confidence we can trust the prediction. Meanwhile, we found that when there is a large visual difference between testing images and training images, the pose precision becomes low. Based on this, we present the dual-level image-similarity strategy (DLISS), which consists of two levels: initial level and iteration-level. The initial level performs feature vector clustering in the training set and feature vector acquisition in testing images. The iteration level, namely, the PSO-based image-block selection algorithm, can select the testing images which are the most similar to training images based on the initial level, enabling us to gain higher pose accuracy in testing set. Our method considers both the accuracy and the robustness of relocalization, and it can operate indoors and outdoors in real time, taking at most 27 ms per frame to compute. Finally, we used the Microsoft 7Scenes dataset and the Cambridge Landmarks dataset to evaluate our method. It can obtain approximately 0.33 m and 7.51${}^{\circ }$ accuracy on 7Scenes dataset, and get approximately 1.44 m and 4.83${}^{\circ }$ accuracy on the Cambridge Landmarks dataset. Compared with PoseNet, our CNN reduced the average positional error by 25% and the average angular error by 27.79% on 7Scenes dataset, and reduced the average positional error by 40% and the average angular error by 28.55% on the Cambridge Landmarks dataset. We show that our multi-task CNN can localize from high-level features and is robust to images which are not in the current scene. Furthermore, we show that our multi-task CNN gets higher accuracy of relocalization by using testing images obtained by DLISS.

## 1. Introduction

The problem of robot relocalization [1] refers to inferring the translation and orientation of a robot from the visual scene representation given only a single image. Robot relocalization is often encountered in many robotic applications, such as augmented reality (AR), mobile robot navigation and simultaneous localization and mapping (SLAM). In SLAM, if the tracking of the robot is lost, global relocalization is started to initial camera’s pose estimation, so the robot can continue to complete its work. In the past several decades, owing to strong interest in this problem, many approaches were developed. We use the theory of technological parasitism [2] to describe these approaches. Since the camera is often fixed on a robot, a main component of vision-based robot relocalization is visual pose estimation in the world coordinate system. Thus, it can be divided into four main types of relocalization methods: measurement-based methods, keyframe-based methods, feature-based methods and learning-based methods [3].

The measurement-based relocalization methods make accurate inferences by using 3d structural information of the scene and try to reproduce 3D pose of the image during shooting. Augmented reality and image 3D reconstruction in SLAM often involve the SFM (structure from motion) [4] which is used to reproduce camera pose.

The keyframe-based methods select the most similar image from collected keyframes and estimate the relative pose. Then, according to the pose of the selected keyframe, it is converted to the global coordinate system to obtain the global pose. In [5,6], algorithms with good performance have been proposed and verified. However, the main limitation of these methods is that they take much time to search for similar keyframes, since the number of keyframes grows rapidly as the robot moves through the environment. Another problem is that due to the sparsity of keyframes, when the similarity between the testing image and the collected keyframes is too low, the accuracy and robustness will be seriously reduced.

The feature-based methods store the feature points extracted from the key-frame image rather than store the keyframes. The corresponding descriptors of image feature points and their positions in global coordinate system are stored as a database. Then, the feature points detected from a new image are matched with the information in the database during relocalization. In general, some robust feature detectors and descriptors can match enough feature points so that many relocalization processes adopt these methods, such as [7,8,9,10]. ORB-SLAM2 algorithm is a fully functional visual SLAM algorithm that is applied to diversified visual sensors. The relocalization in ORB-SLAM2 is realized by a feature matching method which adopts more efficient ORB operators [11] to extract features, making it possible to operate indoors and outdoors in real time. Based on SIFT features [12,13], methods in [14,15] need large databases with sufficient data and efficient retrieval algorithms. They rely heavily on feature detection and feature matching. Therefore, when facing environments with fewer features, motion blur or weak texture, the accuracy of relocalization will decline rapidly, or even fail.

The learning-based methods have shown excellent performances in relocalization in the latest years. The scene coordinate regression forest (SCoRF) proposed by Shotton et al. [9,16] has been successfully applied to camera pose estimation, with taking RGB-D images as input, using depth images to create labels of scene coordinate and mapping each pixel from the camera coordinate system to the global scene coordinate system. Ref. [17] proposed a novel regression forest based visual relocalization method in a coarse-to-fine manner. The work proposed a topological regression tree to predict “coarse” subscenes where the camera is located. There are also some relocalization algorithms using convolutional neural networks. For instance, differentiable sample consensus (DSAC) [18] and DSAC++ [19] were proposed to regress pixel-wise scene coordinates given an input RGB image using deep learning framework. Ref. [20] developed a novel multi-sensor-based indoor global localization system integrating visual localization aided by CNN-based image retrieval with a probabilistic localization approach. The PoseNet algorithm proposed by Kendall et al. [21] is the first algorithm which can use CNN to directly perform regression of camera pose estimation. Adopting the GoogLeNet [22] framework to conduct transfer learning, the algorithm needs images and corresponding global pose during training, while RGB images are only used for global pose estimation. The approach presents a certain robustness in challenging environmental conditions, such as a changing of view angle, motion blur and dim light, which seriously affect feature extraction and matching. The experimental results show that high pose accuracy can still be obtained for the testing set even with a great changes of view angle. In [23], a new loss function based on error of scene reprojection were used to achieve higher efficiency. Esfahani et al. [24], for the first time, designed a new deep network architecture trained by combining deblurring and semantic segmentation modules. Melekhov et al. [25] improved PoseNet by using Hourglass network in place of GoogLeNet with symmetric encoder and decoder network structure for training, and improved the accuracy of relocalization. Jian Wu et al. [26] proposed BranchNet, a multi-task CNN algorithm, which was divided into two branches to predict the translation and rotation of pose respectively, also improving the accuracy of pose estimation to a certain extent. Inspired by RNN [27,28] in the text classification field, some relocalization algorithms based on RNN were proposed. Anh Nguyen et al. [29] first created the event image from a list of events that occur in a very short time interval; then a stacked spatial LSTM network (SP-LSTM) is used to learn the camera pose. Clark et al. [30] used a recursive model to perform pose estimation of video clips, using the GoogLeNet network to extract features from the input images and then transmitting these features into the cell blocks of LSTM [31]. Most of learning-based algorithms adopt a similar CNN structure, extracting features by using a trained model which is trained on large-scale data of image classification, and then returning the pose.

Unlike other three relocalization methods, the pose regression with learning-based methods do not need to store keyframes, match features between frames and perform pose optimization. When exploring large-scale areas with learning-based algorithms, the storage memory and computing time do not increase. Therefore, they can implement large-scale relocalization without area limitations.

Although learning-based relocalization methods can solve many issues in other three relocalization methods, some questions remain unsolved. For instance, ref. [3] found that pose precision is low when there is a large visual dissimilarity between the testing image and the training set. The authors in [3] presented a image cropping algorithm based on a genetic algorithm to select the most similar image within the training set. Besides, ref. [23] proposed that if we input a testing image which is not in the scene of the training set, the algorithm still should output a 6-DOF pose, which is an obviously severe problem that should be solved before applying the model in practice, namely, model uncertainty. Based on this, the authors in [23] proposed Bayesian PoseNet.

In this paper, we present our approach which adopts an end-to-end multi-task CNN for 6-DOF pose estimation and scene recognition by using only RGB images. The scene recognition adopts a simple dichotomy model which detects whether the current image belongs to current scene, and it can solve the problem of model uncertainty. We think using the dichotomy model is more practical, because the robot is only working in one scene, and images from other scenes should be judged not to be in that scene. Experimental results show that scene recognition not only helps the robot to measure the reliability of the predicted pose but also improves the pose precision. At the same time, experimental results show that our multi-task CNN performs better than the method in [23].

**Figure 1:**
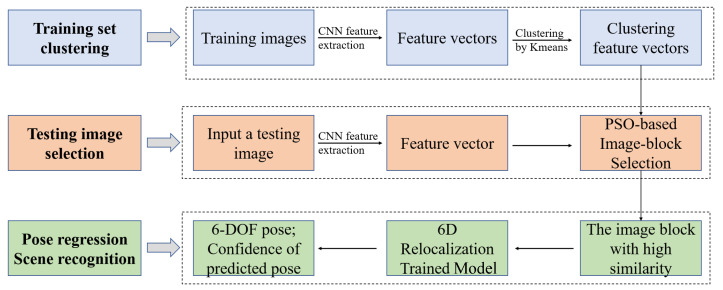
The block-diagram of our methods. The main idea is to crop a testing image into several image blocks, and find the image block with the highest similarity to training set. Firstly, we train our multi-task CNN, thereby obtaining a 6D relocalization trained model. The trained model is used to extract the feature of the image, which forms a feature vector. Secondly, to reduce the computational complexity of image similarity, feature vectors of training images are clustered by k-means. Thirdly, the cropped position of the image, which is regarded as the optimal variable, is optimized by PSO-based image-block selection. Finally, the pose and confidence of predicted pose can be obtained by utilizing 6D relocalization trained model to calculate the selected image block with the highest similarity.

Then, it was found in [3] that if the trajectory of the training set and the trajectory of the testing set are visually similar, the relocalization performance will be better on the testing set. However, the method in [3] has a limitation: the cropped positions of the image cropping algorithm based on genetic algorithm are fixed. Thus, the cropped image may not be the one that is most similar to the training set. Based on this, we propose the PSO-based image-block selection algorithm, using random particle swarm to determine the cropped position. The image similarity between the testing image block and training images is treated as the fitness function. Our CNN is considered as a feature extractor and the extracted feature vectors can be utilized to calculate image similarity. Meanwhile, we found that if we use all training images to calculate image similarity with the testing image, the computational complexity becomes high. We use k-means to cluster feature vectors of training set to reduce the computational complexity, making our network operate in real time. Thus, the Euclidean distances between feature vectors of the testing image and feature vectors of clustering centers are used as measurements of image similarity. Finally, we integrated our methods and propose a preprocessing system of testing images: dual-level image-similarity strategy. After we get the testing image block, we transmit it into 6D relocalization trained model to get the 6DoF pose and the probability that the image belongs to the current scene. We regard the probability as the confidence of the predicted pose. The entire process is shown in Figure 1.

To sum up, we make the following contributions in this paper:

1. We present an end-to-end multi-task CNN which can simultaneously perform 6DoF pose regression and scene recognition tasks by using a single hand-held RGB visual sensor. Compared with the state-art networks, our CNN can not only maintain the stabilization of pose estimation by using the confidence of scene recognition, which may overcome the influence of incorrect scene image on CNN, but also improve the accuracy of 6D relocalization.
2. Besides using multi-task CNN, another contribution on the improvement of relocalization accuracy is that: we present a block selection algorithm for a new input image, which is based on particle swarm optimization to find the most similar block to some training images in the training set.
3. To reduce the computational complexity of finding the most similar image in the whole training set, we adopt k-means, an unsupervised clustering method, to segment the training feature space so as to form clustering feature vectors and then calculate the similarity to the testing image, which can make our model operate in real time.
4. Based on 2 and 3, we present a preprocessing system of testing images, namely, the dual-level image-similarity strategy, which adopts an end-to-end manner to obtain image block most visually similar to training set.

The rest of our paper is organized as follows. In Section 2, we give an introduction to the proposed approaches and datasets used in our experiments. In Section 3, we firstly give the implementation details of our experiments, and then we demonstrate why we can use single CNN to learn both pose regression task and scene recognition task. Finally, we show experimental results of our methods and compare our results with the state-art networks. In Section 4, we present conclusions and some suggestions for future work.

## 2. Proposed Approaches and Datasets

This section introduces the proposed localization approaches for predicting camera pose and datasets used in our experiments separately.

### 2.1. The Specific Methods and Measures

**Figure 2:**
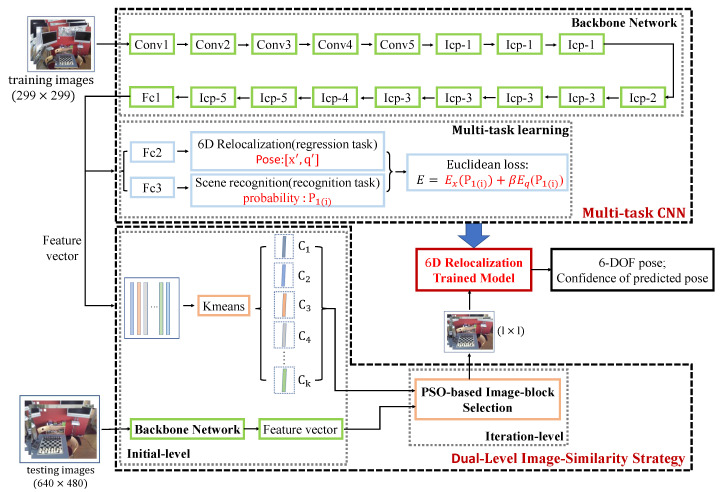
Overview of the proposed relocalization method (taking Chess dataset of 7Scenes as an example). Firstly, for training images, we process the images by center cropping, and then scale them to the size 299 × 299. In training section, we use GoogLeNet Inception V3 as a 6D relocalization backbone network to perform pose regression and scene recognition simultaneously. In testing section, we firstly get the selected testing images through DLISS, which are the most similar to training images. The size of selected images is l × l, and then we resize them to the size 299 × 299. Finally, we transmit them into a 6D relocalization trained model, obtaining the predictive value of pose and the confidence of the predicted pose. The “Icp1-5” in Figure 2 means “Inception1-5” module of GoogLeNet Inception V3.

The whole pipeline of proposed methods is shown in Figure 2, which consists of three modules: the backbone network; multi-task learning for pose regression and scene recognition; and the dual-level image-similarity strategy.

We use GoogLeNet Inception V3 as 6D relocalization backbone network. Then, the trained backbone network is considered as a feature extractor and the extracted feature vectors can be utilized to calculate the image similarity in the DLISS. Meanwhile, we propose multi-task learning, through which the CNN can simultaneously perform pose regression and scene recognition. In pose regression, a fully connected layer Fc2 is added to generate an information-rich feature vector with a dimension of 1024, which finally converges to 6DoF camera pose including position represented by XYZ and orientation represented by quaternion. Similarly, in scene recognition a fully connected layer Fc3 is added to make classification decision. Finally, by minimizing the Euclidean loss, we get the 6D relocalization trained model.

For testing images, we selected the image blocks with the highest similarity to training set via dual-level image-similarity strategy (DLISS), and then we transmit the selected images into a 6D relocalization trained model to get 6DoF pose and the probability that the image belongs to the current scene. In the testing section, we regard the probability as the confidence of predicted pose. We talk more about the backbone network; multi-task learning for pose regression and scene recognition; and DLISS in the system structure.

#### 2.1.1. Backbone Network

When applying the algorithm of deep learning, we usually consider the mainstream network structure as the backbone network, and then retrain it by transfer learning. Widely used as the backbone network in many applications, GoogLeNet achieved higher accuracy than AlexNet and VGG in the ImageNet Challenge, with the number of network parameters greatly reduced and the real-time performance enhanced. GoogLeNet Inception V1 is a 22 layer convolutional network with six “inception modules” and two additional intermediate classfiers which are discarded when testing. Beyond that, the author improved the inception sensing module, introducing GoogLeNet Inception V2, GoogLeNet Inception V3, GoogLeNet Inception V4 and other networks.

The CNN proposed by the paper aims to not only increase the accuracy of relocalization of robots, but also to enable the model to show robustness even in challenging environments. Thus, in this paper, when designing the neural network structure of relocalization, we consider GoogLeNet Inception V3 as the backbone network to extract the features of input images. The network was originally designed for image classification, and the output is the probability of each class. However, the relocalization CNN aims to output 6DoF pose, so the network needs some modifications. The 6DoF pose can be denoted by its 3D location and rotation pose. We chose quaternion to demonstrate the rotation pose, with the simple calculation and without singularity. The output pose which is estimated by our CNN is represented as a 7-dimensional vector *P*:

(1) $$\begin{array}{c} P=[x,q] \end{array}$$

where *x* denotes the corresponding 3-dimensional vector of position for the input image and *q* represents the corresponding 4-dimensional vector of orientation for the input image.

In our methods, we adopt the idea of multi-task learning to enable the model learn two tasks jointly in the CNN, which include the regression task (6D relocalization) and the recognition task (scene recognition).

The recognition task adopts the fully connected layer + softmax to perform scene recognition, namely, a simple dichotomy classification process used to determine whether the image scene is consistent with the robot’s current scene. By minimizing the cross-entropy loss, the softmax classifier gives two probabilities in the ith image, $P_{1(i)}$ and $P_{0(i)}\left(P_{0(i)}+P_{1(i)}=1\right)$, where $P_{1(i)}$ denotes the probability that the image belongs to the current scene and $P_{0(i)}$ demonstrates the probability that the image does not belong to the current scene. In the training process, $P_{1(i)}$ is part of the loss function which can make the loss converge to the minimum. In the testing process, $P_{1(i)}$ is the confidence of the predicted pose which can detect whether the scene is actually present in the input image. Therefore, when the image scene cannot match with the robot’s current scene, the output confidence value will be very low, eliminating the uncertainty of pose estimation and strengthening the robustness of network.

The regression task can generate a representation of pose feature for the image by blending multiple local feature images through fully connected layer, and ultimately use the pose parameters learned by the network to fit the 6DoF pose of the testing images.

Compared with the CNN used for target recognition or image classification, the neural network for 6DoF pose regression employs Euclidean Loss, which is calculated as follows:

(2) $$E=E_{x}+\beta E_{q}$$

(3) $$E_{x}=\frac{1}{batch}\sum_{i=1}^{batch}P_{1(i)}{∥x_{i}^{\prime }-x_{i}∥}_{2}$$

(4) $$E_{q}=\frac{1}{batch}\sum_{i=1}^{batch}P_{1(i)}{∥q_{i}^{\prime }-\frac{q_{i}}{{∥q_{i}∥}^{2}}∥}_{2}$$

where $E_{x}$ denotes the positional error; $E_{q}$ is the angular error; $x_{i}$ and $q_{i}$ a 3D position vector and a quaternion vector representing the orientation in the ith image; $x_{i}^{\prime }$ and $q_{i}^{\prime }$ are the position and orientation obtained by pose regression in the ith image; β is the coefficient of the loss function, which is used to balance the positional error and the angular error. $P_{1(i)}$ is the probability (also the confidence level) that the ith image belongs to the current scene, given by softmax in the scene recognition. During the training of the network for pose estimation, if the accuracy evaluated by the dichotomy classifier for the ith image is high enough, in the loss function of the ith image we will assign high weight to the image. Adversely, for the ith image that does not belong to the current scene, its $P_{1(i)}$ will be so low that its corresponding weights in its loss function will be too small, which will lead optimization function to optimize in a more accurate manner.

#### 2.1.2. Multi-Task Learning for Pose Regression and Scene Recognition

The relocalization refers to the process to reproduce the location and orientation in three dimensions (namely, the camera pose of the images) for a given RGB image. Assuming that the image and its database of camera pose, $D=\left(I,C\right)=\left(i_{1},c_{1}\right),\left(i_{2},c_{2}\right),\cdots ,\left(i_{n},c_{n}\right)$, contains the limiting constraint relation ms for the corresponding image, we can learn the mapping relation between the input image and its pose:

(5) $$\begin{array}{c} c_{i}=f_{ms}\left(i\right) \end{array}$$

Define a test sample $\left(i^{\prime },c^{\prime }\right)$, where $c^{\prime }$ is the camera pose of the sample, and $i^{\prime }$ is the image of the sample. Learning the mapping relation is equivalent to learning the distribution of conditional probability $P(c^{\prime }|i^{\prime })$. In fact, the model learns the posterior information of the input image after absorbing the training set *D*, and then uses the posterior information to predict what pose corresponding to $i^{\prime }$ it should output. Thus, the dataset *D* is also one condition for the conditional probability $P(c^{\prime }|i^{\prime })$.

(6) $$\begin{array}{c} P\left(c^{\prime }|i^{\prime }\right)=P\left(c^{\prime }|i^{\prime },D\right) \end{array}$$

The model learns from the dataset *D*, absorbing the information about the structure of the scene and the information about the projection process to form the parameter θ of the model. That means the intention of using dataset *D* is to form θ, so the probability of the pose of input images is as follows:

(7) $$\begin{array}{c} P\left(c^{\prime }|i^{\prime }\right)=P\left(c^{\prime }|i^{\prime },D\right)=P\left(c^{\prime }|i^{\prime },\theta \right) \end{array}$$

Therefore, computing the $P(c^{\prime }|i^{\prime })$ from the $P(c^{\prime }|i^{\prime },\theta )$ means learning the posterior distribution of the theta given the training set *D*, namely, P(θ|D), and then $P(c^{\prime }|i^{\prime })$ can be represented by the following formula:

(8) $$\begin{array}{c} P\left(c^{\prime }|i^{\prime }\right)=P\left(c^{\prime }|i^{\prime },D\right)=\int P\left(c^{\prime },\theta |i^{\prime },D\right)d\theta =\int P\left(c^{\prime }|i^{\prime },\theta \right)P\left(\theta |D\right)d\theta \end{array}$$

With the above derivation, the problem of finding the conditional probability of the test sample is converted to the problem of solving the posterior distribution P(θ|D) of the model parameter theta. Using Bayesian formula to transform the posterior distribution of the model into the following formula:

(9) $$\begin{array}{c} P\left(\theta |D\right)=\frac{P(C|I,\theta )P(\theta )}{P(C|I)}=\frac{P(C|I,\theta )P(\theta )}{\int P(C|I,\theta )P(\theta )d\theta } \end{array}$$

where P(C|I) is the conditional probability distribution of pose estimation given the images in training set; *P*(*C*|*I*,θ) denotes the likelihood function of P(C|I) on *D*. P(θ) represents the prior distribution. Therefore, we finally get the following formula to get the conditional probability of the output pose *c*${}^{\prime }$ given the input image *i*${}^{\prime }$:

(10) $$\begin{array}{c} P\left(c^{\prime }|i^{\prime }\right)=\int P\left(c^{\prime }|i^{\prime },\theta \right)P\left(\theta |D\right)d\theta =\int P\left(i^{\prime },c^{\prime }|\theta \right)\frac{P(C|I,\theta )P(\theta )}{\int P(C|I,\theta )P(\theta )d\theta }d\theta \end{array}$$

Understanding all the derivations, the pose estimation is converted to solve the maximum posterior distribution P(θ|D) of the model.

Essentially, the regression network is used to learn the posterior distribution model P(θ|D) of the dataset. Based on the model θ, the output of the regression network is to predict the mean value of the conditional distribution of the 6DoF pose. As we define the Euclidean loss, the output pose of the network is only approximate to the conditional mean value of pose, which is defined by the training set. The use of Euclidean loss assumes that the error distribution of the model parameters obeys Gaussian distribution. Therefore, the traditional CNN for relocalization can be regarded as predicting the mean value u of a single Gaussian conditional distribution of poses:

(11) $$\begin{array}{c} P\left(c^{\prime }|i^{\prime }\right)=P\left(\left[x,q\right]|D\right)=N\left(\mu ,\sigma^{2}\right) \end{array}$$

However, it is hard to satisfy such an assumption. When the input image is not in the scene of the training set, the model can not estimate the image’s accurate location, namely, the model uncertainty. If we did input said image in the pose regression model, the model would still output a pose, which is an obvious severe problem that should be solved before applying the model in practice.

One practicable solution is to use a Gaussian mixture model in place of a single Gaussian model, by modeling for the uncertainty and establishing the distribution of posterior pose for multi models. In this paper, we propose to add a task for scene recognition, a dichotomy classification module used to determine whether the input image is in the current scene, and we use the recognition results to measure the model uncertainty. This method is essentially a two-mode Gaussian model to solve the uncertainty problem.

#### 2.1.3. Dual-Level Image-Similarity Strategy

In general, visual pose regression algorithm based on deep learning requires images and corresponding poses to train network parameters. Then, we use the trained network to perform pose regression on the testing set. We think that the pose accuracy is higher when the trajectory of the testing set is closer to the training set.

In our CNN, the size of input images should be 299 × 299, and we need to process images before training. We process images of training set by center cropping, and then scale them to the size 299 × 299. We can replicate the process for images in testing set, but in practice, it is found that by the cropping method, we can gain images which are the most similar to training images, making the accuracy of pose regression higher. Thus, we propose dual-level image-similarity strategy, which consists of initial level and iteration level.

##### Initial Level

In this paper, GoogLeNet Inception V3 is used as the backbone network with a 2048-dimensional fully connected layer as the final layer. Therefore, the 2048-dimensional (2048-d) feature vector outputted by the fully connected layer can be used as the feature vectors of images to measure the image similarity. Then we calculate the distance between the feature vectors of testing images and the feature vectors of training set, and we choose the test image which has the minimum distance with training set as the input of our network.

However, there are usually too many images in the training set. For example, in Microsoft’s 7Scenes dataset, there are thousands of images for each scene. Moreover, the feature vector’s dimension is relatively high, which takes much time and effort to calculate directly. In order to lower the complexity of calculation, we adopt k-means to cluster the feature vectors of the training set and obtain some clustering centers {$C_{1}$,$C_{2}$...$C_{k}$}. The feature vector of every clustering center is also a 2048-d feature vector. Essentially, clustering the feature vectors of the training set is unsupervised clustering of camera trajectory. Then, the distance between the feature vectors of testing set and the clustering centers is used as a measurement for image similarity. To obtain better clustering performance, the training set is standardized before clustering.

(12) $$\begin{array}{c} \vec{u_{i}^{\prime }}\left(j\right)=\left\{\begin{array}{c} \frac{\vec{u_{i}}\left(j\right)-u\left(j\right)}{\delta (j)},\delta \left(j\right)\neq 0\\ 0,\delta (j)=0 \end{array}\right.\left(i\in \left[1,m\right],j\in \left[1,s\right],i,j\in N^{+}\right) \end{array}$$

where *u* denotes the mean of feature vectors of training set, δ represents the standard deviation, *s* is the number of dimensions and $u_{ij}$ is the *j*th dimension of the *i*th feature vector after standardization.

For testing images, we firstly resize them to the size 299 × 299, and we transmit them into the backbone network, getting 2048-d feature vectors. Similarly, in PSO-based image-block selection algorithm, when we get the selected images, we use the same method to get 2048-d feature vectors.

Through feature vector clustering in the training set, we obtain clustering centers {$C_{1}$,$C_{2}$…$C_{k}$}. Each clustering center can be represented by a 2048-d feature vector. After we get feature vectors $C_{test}$ of testing images, the Euclidean distances between the feature vector of testing images and the clustering center are used as measurements for image similarity.

(13) $$E_{distance}={∥C_{k}-C_{test}∥}_{2}$$

##### Iteration-Level: PSO-Based Image-Block Selection Algorithm

We present a block selection algorithm for a new input image, namely, PSO-based image-block selection algorithm, which is based on particle swarm optimization to find the most similar block to some training images in the training set.

The particle swarm optimization (PSO) [32] is a swarm intelligence method that models social behavior to guide swarms of particles towards the most promising regions of the search space. It designs a massless random particle and finds the optimal solution through iteration. A particle has only two properties: velocity and location. Velocity represents how fast or slow it is moving, and location represents the direction it is moving in. Each particle searches for the optimal solution separately in the search space, and records it as the current individual extreme value, then shares the individual extreme value with other particles in the whole particle swarm, and finds the optimal individual extreme value as the current global optimal solution of the whole particle swarm. All particles in the particle swarm adjust their velocity and location according to the current individual extreme value found by themselves and the current global optimal solution shared by the whole particle swarm.

It is easily implemented, and it usually results in faster convergence rates than the genetic algorithms [33]. Meanwhile, we find that [3] using the image cropping algorithm based on genetic algorithm to select the most similar image with training set has some limitation: the cropped positions are fixed. According to the method presented in [3], we know that if an image is 640 × 480 in size, it has only 160 cropped positions, which results that the cropped image may not be the one that is most similar to the training set. However, the proposed PSO-based image-block selection algorithm uses random particle swarm to determine the cropped position, and the particle swarm can traverse all the positions in the image, which means the cropped position can be at any pixel of the image. Thus, our algorithm will be more excellent and robust.

The PSO-based image-block selection algorithm’s steps are as follows.

Initialize Particle Swarm. We initialize two properties of the particle swarm, its location and velocity. The location property contains four parameters for each particle, with $x_{i}$ and $y_{i}$ representing the upper-left coordinates of the selected images given by testing set, $l_{i}$ representing the edge length of the selected images and $s_{i}$ representing the distance which is used to measure the level of image similarity between the feature vectors of selected images and the clustering center of training set. For each particle, its four parameters of the velocity property are set to 0.

Update the Formula of Velocity and Location. We update the location and velocity of the particle by the following formula.

(14) $$v_{i}=w\times v_{i}+c_{1}\times rand\left(\right)\times \left(pbest_{i}-o_{i}\right)+c_{2}\times rand\left(\right)\times \left(gbest-o_{i}\right)$$

(15) $$o_{i}=o_{i}+v_{i}$$

where *w* is called inertia factor, with its value non-negative. $c_{1}$ and $c_{2}$ are called the accelerating constants. rand() represents the random number of the interval [0,1]. $pbest_{i}$ refers to the individual extremum, which finds the optimal solutions for each particle. gbest refers to the global optimal solution, which is the best one optimal solution among all the optimal solutions of each particle.

Update Individuals’ Extreme Value and Global Optimal Solution. We use the distance between the feature vectors of images in testing set and the clustering center of the training set as the measurement of image similarity. The smaller the distance obtained is, the more similar the two are and the higher the $s_{i}$ is. When the $s_{i}$ obtained from a particle is higher than the $s_{i}$ of the particle’s individual extreme value, we take the four parameters of the current particle during the iteration as the new pbest. Similarly, when the $s_{i}$ obtained is higher than the $s_{i}$ in the global optimal solution, we take the four parameters of the particle during the iteration as the new gbest.

Set distance threshold $d_{threshold}$. It is found that if the testing image and the clustering centers of training set are in the same scene, the distance between them will be much lower than if they are not. Thus, we set a distance threshold to prevent our algorithm from processing testing images not in the current scene, which makes our algorithm more robust.

**Figure 3:**
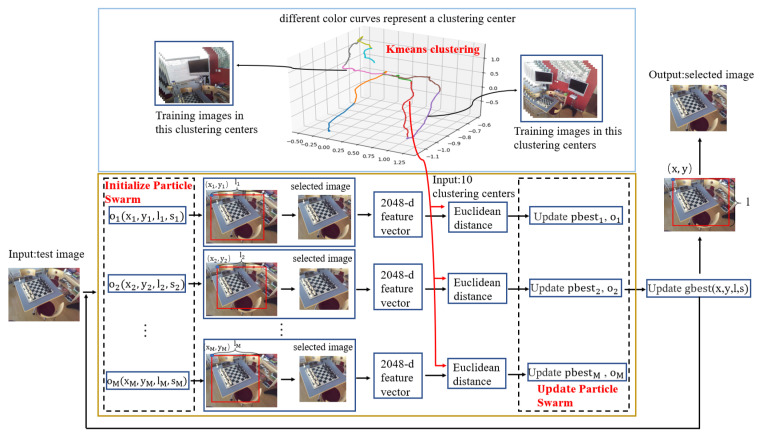
The flow block diagram of the PSO-based image-block selection algorithm. Using this method, the image which is most similar to the training set can be selected.

In Figure 3, we show how to get the selected images in detail through PSO-based image-block selection. Pseudocode of the algorithm is shown in the Algorithm 1.
**Algorithm 1:** PSO-based image-block selection.
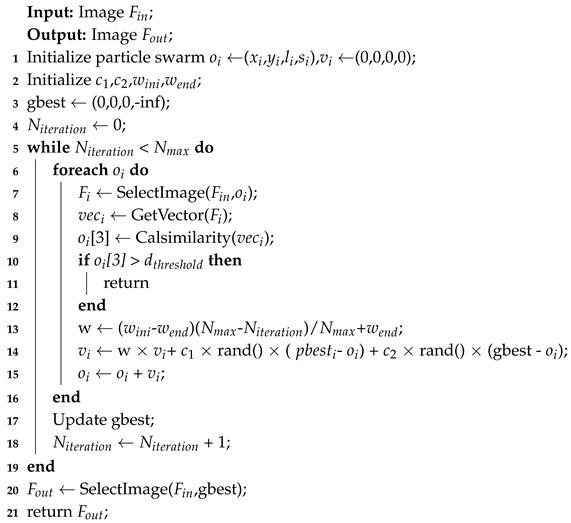


### 2.2. Datasets

We evaluate the proposed approach on two different datasets for camera relocalization.

7Scenes. The indoor dataset 7Scenes, which is recorded using a RGB-D camera at a resolution of 640 × 480 pixels and published by Microsoft Research, includes 7 indoor scenes and is used for image-based localization [21,23,26]. The dataset exhibits shape and color ambiguities and motion blur, which is extremely challenging for purely visual relocalization using feature-based methods.

Cambridge Landmarks. The vision team of the University of Cambridge release an outdoor urban localization dataset with 5 scenes. The dataset provides data to train and test pose regression algorithms in a large scale outdoor urban setting.

In our experiments we utilize the same train and test datasets for each scene as provided in the original two datasets.

## 3. Experiments

In this section, we firstly give the implementation details of our experiments, and then we demonstrate why we can use single CNN to learn both pose regression task and scene recognition task. Finally, we show experimental results of our methods and compare our results with the state-art networks, such as the PoseNet [21] and Bayesian PoseNet [23].

### 3.1. Implementation Details

#### 3.1.1. Training Details for 6D Relocalization Network

During training, tensorflow of Google is used and stochastic gradient descent is adopted. The scale factor β in the loss function (2) was set to 18. The initial learning rate was set to $10^{-5}$ and dropped by 90% every 2000 iterations. Training was end at 24,000 iterations. Using an NVIDIA TITAN RTX which is produced by YUEHONG in Harbin, China, training took about 5 h with the batch size of 128.

**Figure 4:**
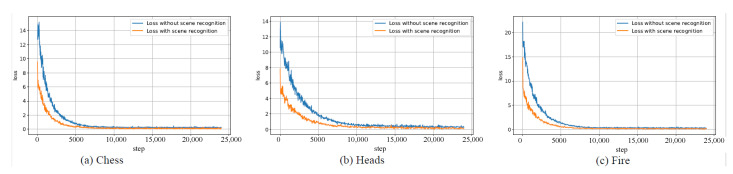
Relocalization loss of two networks.This demonstrates that the network with scene recognition is better than the network without scene recognition.

We did a comparison experiment to prove the effectiveness of scene recognition in the training process. We use transfer learning algorithm to respectively train the two 6D relocalization networks in which one is attached with scene recognition function and the other is not. When we train the network with scene recognition, we add images of other scenes to enhance the robustness of the network. Figure 4 shows relocalization loss of the two networks in Chess, Heads, Fire. From Figure 4, we can see that the loss of 6D relocalization network with scene recognition decreases faster and converges faster, which illustrates the scene recognition can improve pose accuracy.

#### 3.1.2. Initialization of PSO-Based Image-Block Selection

For the training images, we first crop and then compress them to get the size 299 × 299 needed by the CNN.

For the testing images, using the parameters in Table 1, we pick out the images with the size l × l through PSO-based image-block selection algorithm, and then the images will be scaled to 299 × 299.

### 3.2. Feature Representation in Pose Regression and Scene Recognition

**Figure 5:**
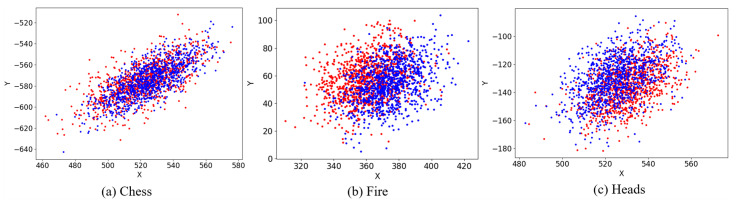
The projection points on the xoy plane. The red points represent the outputs given by the first layer of GoogLeNet Inception V3, which is responsible for pose regression, and the blue points represent the outputs given by the last layer of GoogLeNet Inception V3, which is responsible for image recognition.

We take a sequence in the Chess, Fire and Heads dataset of 7Scenes to illustrate why we can use one backbone CNN for both pose regression and scene recognition. We use two GoogLeNet Inception V3 networks to analyze the problem, one of which is responsible for image pose regression while the other is used for image recognition. Two networks can output their own corresponding feature vectors respectively through their last layer, and we use t-SNE to visualize the two output vectors in Figure 5. In Figure 5a–c, we get the projection points on the two-dimensional plane, which are corresponding to the outputs given by the last layer of the two networks, and it is found that the overall convergence position of the projection points is almost the same, suggesting two networks learn almost the same feature representation in the low-level layers. This further indicates that the CNN which is used for classification will always store the pose information until the last layer, no matter whether it will be expressed in the output, which illustrates that it is feasible to use single CNN to learn both pose regression task and scene recognition task.

According to our analysis in Section 2 (Backbone Network), we know that if the scene of the input image is not in the training set, the network will output a very low confidence for the predicted pose and the outcome of relocalization is not convincing. Therefore, we can be sure that training the network with scene recognition can not only help to solve the model uncertainty problem, but also improve the accuracy of pose estimation.

### 3.3. Results of Dual-Level Image-Similarity Strategy

#### 3.3.1. Feature Vector Clustering

To get high pose precision, we get the selected testing images by DLISS. To lower the complexity of later matrix calculation, we use the k-means algorithm to cluster, and then calculate the distances between clustering centers and feature vectors to measure the image similarity between testing images and training images. In our experiments, since the number of images in training set was different, we set different numbers of clustering centers, which correspond to the numbers of images, shown in Table 2.

**Figure 6:**
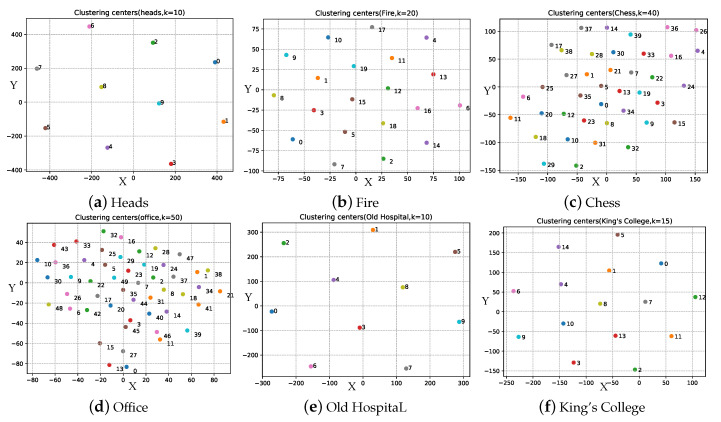
Visualization of clustering centers (after dimensional reduction by t-SNE). We can see that the clustering centers are evenly distributed, showing the high effectiveness of clustering.

In order to observe the effect of clustering, t-SNE, a dimensionality reduction algorithm for high dimensional data is used to reduce the dimension of the 2048-dimensional vector of clustering center, with the vectors reduced to two-dimensional vector [X,Y] which can be displayed on a two-dimensional plane. As shown in Figure 6, for four datasets of 7Scenes and two datasets of the Cambridge Landmarks Dataset, their 2-dimensional distributions are uniform, which are gained by reducing the dimensions of feature vector of clustering centers, showing the high effectiveness of clustering.

**Figure 7:**
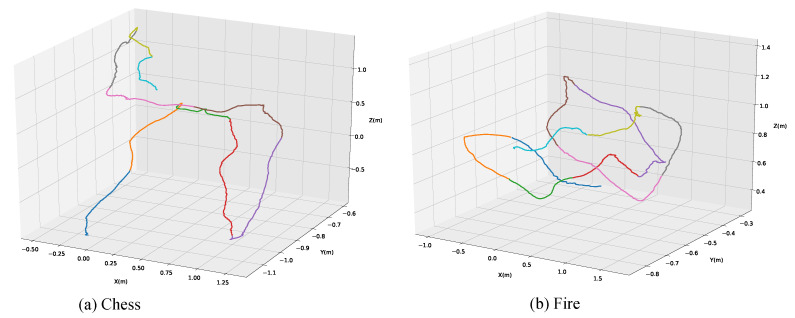
Camera trajectory in Chess scene(seq-01) and Fire scene(seq-01). We use k-means for unsupervised clustering of camera trajectory. The different colored curves in the figure represent clustering centers.

After clustering the feature vectors of training set, we show camera trajectory in the Chess scene(seq-01) and Fire scene(seq-01) in Figure 7. For Chess scene(seq-01) and Fire scene(seq-01) in Figure 7, we can see that the camera trajectory consists of 10 different color curves, each curve representing a clustering center. It indicates that k-means can segment the training feature space uniformly, which illustrates the effectiveness of reducing complexity of calculation through clustering.

#### 3.3.2. The Robustness of the PSO-Based Image-Block Selection Algorithm

**Figure 8:**
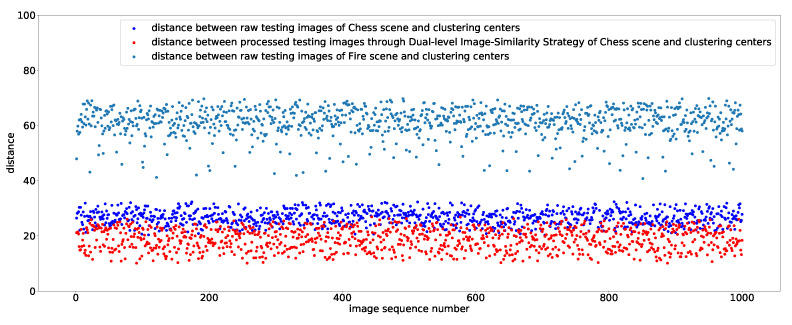
The distance between feature vectors of three types of testing images and clustering centers of the training images in Chess scene.

The Euclidean distance is used as a measurement of image similarity to get the image which is most similar to the nearest neighbor training image. Figure 8 shows the distance between three types of testing images and clustering centers of the training images in Chess scene. Figure 8 indicates that the distance between testing images of Chess scene and clustering centers of Chess scene becomes close, which shows that through DLISS we can always get the image block with the highest similarity to training set. Meanwhile, for testing images of Fire scene, the distance between most testing images and clustering centers of Chess scene exceeds the distance threshold, so the proposed algorithm will not process these images, which makes algorithm more robust. In Figure 8, we can see that there are still some testing images that have not been eliminated by the algorithm, and these images will be sent to the network for pose regression. However, the scene recognition of our multi-task CNN will give a particularly low confidence for the predicted pose of these images, which indicates that our algorithm and scene recognition can solve the problem of model uncertainty together.

#### 3.3.3. The Reliability of the Dual-Level Image-Similarity Strategy

**Figure 9:**
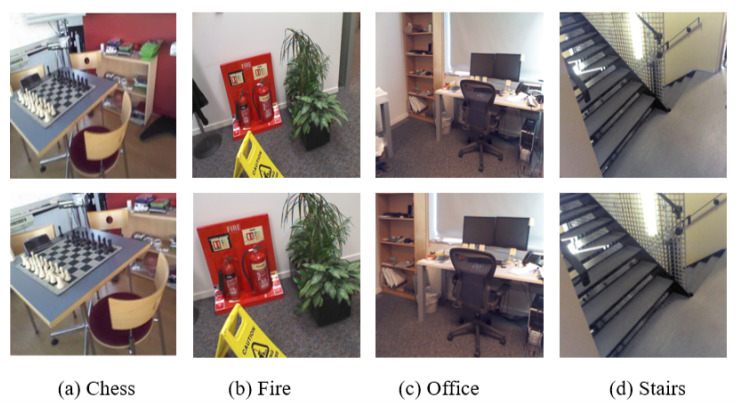
The testing images (above) obtained by center cropping and zooming; selected images (below) obtained by DLISS. The positional error and angular error of these images are as follows: (**a**) above: 0.283 m, 3.965${}^{\circ }$ below: **0.275 m, 3.951${}^{\circ }$**; (**b**) above: 0.384 m, 12.318${}^{\circ }$ below: **0.366 m, 9.261${}^{\circ }$**; (**c**) above: 0.403 m, 6.732${}^{\circ }$ below: **0.371 m, 6.511${}^{\circ }$**; (**d**) above: 0.311 m, 7.874${}^{\circ }$ below: **0.296 m, 7.276${}^{\circ }$**. We prove that DLISS can reduce the positional error and angular error in testing images.

To illustrate the effectiveness of the DLISS, we choose 4 selected images which have relative long distance with the clustering centers of training images, and use them for comparison experiment. The long distance with the clustering centers means that the cropped images have low image similarity with clustering centers. We process testing images above by center cropping and resize them to the size 299 × 299. For the selected images by DLISS, the size of the images is l × l, and we resize the images to 299 × 299. As shown in Figure 9, the selected images retain the scene structure information of the raw testing images. We can see that through DLISS we can get lower positional error and angular error. We think this is because that the camera trajectory of testing images and the camera trajectory of training images become closer after DLISS. In Section 3.4, we will prove the validity of DLISS on the whole testing set of 7Scenes and the Cambridge Landmarks dataset.

### 3.4. Experimental Results and Discussion

We conducted the experiments respectively in 7scenes dataset and the Cambridge Landmarks Dataset, and then compared our methods with the PoseNet [21] and Bayesian PoseNet [23], the traditional CNN for relocalization. The experimental results are shown in Table 2.

The results of “Our methods without scene recognition” were gained by transmitting the testing images which were selected by DLISS to the trained 6D relocalization network without scene recognition. The results of “Our methods without using DLISS” were gained by transmitting the testing images which were gotten by center cropping and zooming the raw testing images to the trained 6D relocalization network with scene recognition. The results of “Our methods” were gained by transmitting the testing images which were selected by DLISS to the trained 6D relocalization network with scene recognition.

From Table 2, it is found that after adding the scene recognition module, the positional error and the angular error are reduced. Meanwhile, by comparing the results of “Our methods” and “Our methods without using DLISS,” we can see that using testing images obtained by DLISS can improve the accuracy of pose regression. The difference between “Our methods” and “Our methods without using DLISS” is only the source of testing images. The difference between “Our methods” and “Our methods without using scene recognition” is only whether there is scene recognition in the network.

In Table 3, we show the number of images in other scenes added when training the network with scene recognition and the accuracy of scene recognition.

**Figure 10:**
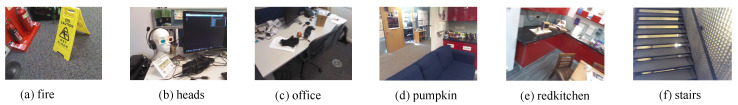
When we performed relocalization in Chess scene, the scene recognition module determined whether the input image belonged to Chess scene, giving the confidence of predicted pose from left to right: 12.3%, 16.5%, 14.4%, 23.3%, 18.6%, 16.6%.

Figure 10 shows that the confidence of predicted pose given by scene recognition to images from other scenes when we perform relocalization in Chess scene. We can see that the confidence given by scene recognition to these pictures is particularly low, so the predicted pose of these pictures by CNN will not be credible, which indirectly indicates that our CNN is robust. Further, as can be seen from Table 3 that scene recognition module will make our CNN loss the ability to localize some images in Chess scene since the accuracy of scene recognition is not 100%, but it will prevent network from localizing images of other scenes, which makes our CNN more robust and also solve the uncertainty of the network.

**Figure 11:**
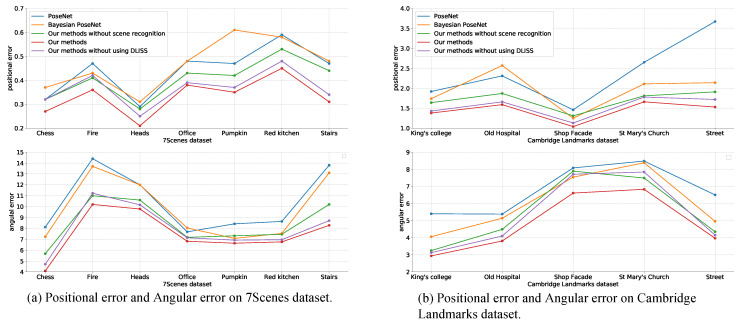
Comparison of the 7Scenes dataset and the Cambridge Landmarks dataset. This shows that our network performs better than PoseNet and Bayesian PoseNet.

We compare the positional error and angular error of the five methods in Table 2, with the comparison outcome showing in Figure 11. The experimental outcomes show that both the positional error and angular error gained by our methods reduced. Compared with PoseNet, our methods reduced the average positional error by 25% and reduced the average angular error by 27.79% on the 7Scenes dataset, and reduced the average positional error by 40% and reduced the average angular error by 28.55% on the Cambridge Landmarks dataset.

**Figure 12:**
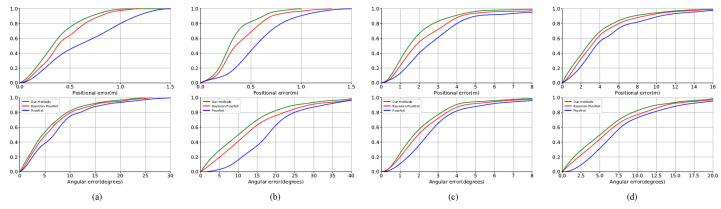
Localization performance from left to right: (**a**) pumpkin, (**b**) stairs, (**c**) King’s College, (**d**) St Mary’s Church. These figures show our localization accuracy for both position and orientation as a cumulative histogram of errors for the entire testing set.

To illustrate the effects of our methods in detail, we take the Pumpkin, Stairs dataset in 7Scenes and King’s College, St Mary’s Church in the Cambridge Landmarks dataset as examples to do a comparative analysis between the network proposed in this paper and the PoseNet. Figure 12 shows the error accumulation histograms of our methods, PoseNet and Bayesian PoseNet on the whole testing set respectively. This shows that our methods perform consistently better than PoseNet and Bayesian PoseNet for all error thresholds. In the error accumulation histograms, the closer the curve is to the left, the smaller the error distribution of the method represented by this curve is.

Use the Pumpkin dataset and King’s College dataset as examples to quantitatively analyze the superiority of our methods. With the total 2000 images in testing set of pumpkin dataset and inputting them into the network frame by frame, we can conduct the relocalization experiment respectively and calculate the positional error and the angular error, the differences between the estimated values and the ground truth. It can be seen from Figure 12a that the positional accuracy of our methods is much better than that of PoseNet and Bayesian PoseNet in terms of both the location and the direction. In this paper, for our methods, 76% of positional error is less than 0.5 m and 83% of the angular error is less than 10${}^{\circ }$. In addition, in terms of large errors, no positional error of our CNN is greater than 1 m while for the PostNet, 20% of positional error exceeds 1 m. Similarly, no angular error of our algorithm exceeds 25${}^{\circ }$ while for the PoseNet, 4% of the angular error is greater than 25${}^{\circ }$.

In the King’s College dataset, our methods obtains approximately 1.46 m and 2.94${}^{\circ }$ accuracy. From Figure 12c, we can see that 67% of positional error is less than 2 m and 90% of the angular error is less than 4${}^{\circ }$. In addition, in terms of large errors, only 8% positional error of our methods is greater than 4 m while for the PostNet, 10% of positional error exceeds 5 m. Similarly, only 3% angular error of our methods exceeds 5${}^{\circ }$ while for the PoseNet, 8% of the angular error is greater than 5${}^{\circ }$.

### 3.5. Efficiency of Our Network

In this paper, we adopt GoogLeNet Inception V3 as the backbone network. Storing the weights took 93 MB for our CNN. We tested our CNN with an NVIDIA Geforce GTX 1060, and reached a speed of 37 fps, which satisfies the real-time requirement in many robotic applications.

## 4. Conclusions

In this study, we propose visual 6D relocalization techniques based on a multi-task CNN. Our CNN can simultaneously perform pose regression task and scene recognition tasks. Experiments show that scene recognition can improve the accuracy and robustness of relocalization. Besides, to further improve the accuracy of relocalization, we present the dual-level image-similarity strategy to select the most similar block in a testing image to the training set. In our strategy, we use k-means for unsupervised clustering of camera trajectory when measuring the image similarity between the testing image and training images, which reduces the computational complexity and makes our model operate in real time. Experimental results show that all of the above techniques improve relocalization accuracy significantly.

One limitation of our method (and PoseNet methods [21,23]) is that it only applies to scenarios where depth information is unavailable, since when depth information is available, there are more accurate methods, such as SCoRe forests [16]. How to use the depth information to improve the performance of CNN is still a problem to be solved. Meanwhile, Although our CNN takes 27 ms per frame to estimate the pose, which can be considered as real-time performance as in PoseNet [21], it still may not fast enough for high-speed robotic applications. Therefore, another interesting issue is to research the compact network architecture that can achieve competitive pose relocalization results while having fewer layers and parameters, which could improve the speed of the the network and allow it to be used in more realistic scenarios. Furthermore, we will keep improving the proposed methods in this paper to generalize their relocalization abilities in unseen scenarios and deploy them on a robot to achieve full SLAM. 

## Figures and Tables

**Table 1:** Initializing the parameters for PSO-based image-block selection.

Operating Parameters	Descriptions	Values
M	Scale of particle swarm	M = 30
$w_{ini}$	Initial inertia weight	$w_{ini}$ = 0.9
$w_{end}$	Inertia weight after maximum iteration	$w_{end}$ = 0.4
$c_{1}$, $c_{2}$	Accelerating constants	$c_{1}$ = 2, $c_{2}$ = 2
$N_{max}$	Maximum number of iteration	$N_{max}$ = 200
$d_{threshold}$	Distance threshold	$d_{threshold}$ = 50

**Table 2:** Dataset details and results. We can see that our CNN show better performance on all scenes than PoseNet and Bayesian PoseNet. We also can see that the positional error and the angular error are reduced with the scene recognition.

Dataset	Train	Test	Clustering	PoseNet	Bayesian	Our Methods	Our Methods	Our
	Frames	Frames	Centers K		PoseNet	without Using	without	Methods
						Scene Recognition	Using DLISS	
Chess	4000	2000	40	0.32 m, 8.12${}^{\circ }$	0.37 m, 7.24${}^{\circ }$	0.32 m, 5.68${}^{\circ }$	0.32 m, 4.71${}^{\circ }$	**0.27 m, 4.1${}^{\circ }$**
Fire	2000	2000	20	0.47 m, 14.4${}^{\circ }$	0.43 m, 13.7${}^{\circ }$	0.41 m, 11.0${}^{\circ }$	0.42 m, 11.23${}^{\circ }$	**0.36 m, 10.2${}^{\circ }$**
Heads	1000	1000	10	0.29 m, 12.0${}^{\circ }$	0.31 m, 12.0${}^{\circ }$	0.28 m, 10.6${}^{\circ }$	0.25 m, 10.16${}^{\circ }$	**0.21 m, 9.78${}^{\circ }$**
Office	6000	4000	50	0.48 m, 7.68${}^{\circ }$	0.48 m, 8.04${}^{\circ }$	0.43 m, 7.18${}^{\circ }$	0.39 m, 7.14${}^{\circ }$	**0.38 m, 6.82${}^{\circ }$**
Pumpkin	4000	2000	40	0.47 m, 8.42${}^{\circ }$	0.61 m, 7.08${}^{\circ }$	0.42 m, 7.32${}^{\circ }$	0.37 m, 6.92${}^{\circ }$	**0.35 m, 6.64${}^{\circ }$**
Red Kitchen	7000	5000	50	0.59 m, 8.64${}^{\circ }$	0.58 m, 7.54${}^{\circ }$	0.53 m, 7.46${}^{\circ }$	0.48 m, 6.96${}^{\circ }$	**0.45 m, 6.76${}^{\circ }$**
Stairs	2000	1000	20	0.47 m, 13.8${}^{\circ }$	0.48 m, 13.1${}^{\circ }$	0.44 m, 10.2${}^{\circ }$	0.34 m, 8.71${}^{\circ }$	**0.31 m, 8.28${}^{\circ }$**
Average				0.44 m, 10.4${}^{\circ }$	0.47 m, 9.81${}^{\circ }$	0.40 m, 8.49${}^{\circ }$	0.37 m, 7.98${}^{\circ }$	**0.33 m, 7.51${}^{\circ }$**
King’s college	1220	343	15	1.92 m, 5.40${}^{\circ }$	1.74 m, 4.06${}^{\circ }$	1.64 m, 3.25${}^{\circ }$	1.43 m, 3.13${}^{\circ }$	**1.38 m, 2.94${}^{\circ }$**
Street	3015	2923	30	3.67 m, 6.50${}^{\circ }$	2.14 m, 4.96${}^{\circ }$	1.91 m, 4.35${}^{\circ }$	1.72 m, 4.15${}^{\circ }$	**1.53 m, 3.97${}^{\circ }$**
Old Hospital	895	182	10	2.31 m, 5.38${}^{\circ }$	2.57 m, 5.14${}^{\circ }$	1.87 m, 4.49${}^{\circ }$,	1.66 m, 4.10${}^{\circ }$	**1.59 m, 3.81${}^{\circ }$**
Shop Facade	231	103	10	1.46 m, 8.08${}^{\circ }$	1.25 m, 7.54${}^{\circ }$	1.31 m, 7.89${}^{\circ }$	1.13 m, 7.71${}^{\circ }$	**1.04 m, 6.61${}^{\circ }$**
St Mary’s Church	1487	530	15	2.65 m, 8.48${}^{\circ }$	2.11 m, 8.38${}^{\circ }$	1.81 m, 7.49${}^{\circ }$	1.78 m, 7.84${}^{\circ }$	**1.66 m, 6.83${}^{\circ }$**
Average				2.40 m, 6.76${}^{\circ }$	1.96 m, 6.02${}^{\circ }$	1.71 m, 5.49${}^{\circ }$	1.54 m, 5.38${}^{\circ }$	**1.44 m, 4.83${}^{\circ }$**

**Table 3:** The number of images in other scenes added and the accuracy of scene recognition.

Dataset	The Number of	Scene
	Images in Other Scenes	Recognition Accuracy
Chess	2000	96.63%
Fire	1000	96.31%
Heads	500	91.69%
Office	3000	93.81%
Pumpkin	2000	86.41%
Red Kitchen	3500	87.53%
Stairs	1000	98.41%
King’s college	600	84.23%
Street	1200	87.56%
Old Hospital	400	86.68%
Shop Facade	100	84.71%
St Mary’s Church	700	85.68%
